# Quantitative CT Evaluation of Small Pulmonary Vessels Has Functional and Prognostic Value in Pulmonary Hypertension

**DOI:** 10.1148/radiol.210482

**Published:** 2022-07-12

**Authors:** Yousef Shahin, Samer Alabed, Dheyaa Alkhanfar, Juerg Tschirren, Alex M. K. Rothman, Robin Condliffe, James M. Wild, David G. Kiely, Andrew J. Swift

**Affiliations:** From the Department of Infection, Immunity and Cardiovascular Disease (Y.S., S.A., D.A., A.M.K.R., J.M.W., D.G.K., A.J.S.) and INSIGNEO, Institute for in silico Medicine (D.G.K., A.J.S.), University of Sheffield, Glossop Rd, Sheffield S10 2JF, England; Department of Clinical Radiology, Sheffield Teaching Hospitals, Sheffield, England (Y.S., S.A., A.J.S.); VIDA Diagnostics, Coralville, Iowa (J.T.); and Sheffield Pulmonary Vascular Disease Unit, Royal Hallamshire Hospital, Sheffield, England (R.C., D.G.K.).

## Abstract

**Background:**

The in vivo relationship between peel pulmonary vessels, small pulmonary vessels, and pulmonary hypertension (PH) is not fully understood.

**Purpose:**

To quantitatively assess peel pulmonary vessel volumes (PPVVs) and small pulmonary vessel volumes (SPVVs) as estimated from CT pulmonary angiography (CTPA) in different subtypes of PH compared with controls, their relationship to pulmonary function and right heart catheter metrics, and their prognostic value.

**Materials and Methods:**

In this retrospective single-center study performed from January 2008 to February 2018, quantitative CTPA analysis of total SPVV (TSPVV) (0.4- to 2-mm vessel diameter) and PPVV (within 15, 30, and 45 mm from the lung surface) was performed.

**Results:**

A total of 1823 patients (mean age, 69 years ± 13 [SD]; 1192 women [65%]) were retrospectively analyzed; 1593 patients with PH (mean pulmonary arterial pressure [mPAP], 43 mmHg ± 13 [SD]) were compared with 230 patient controls (mPAP, 19 mm Hg ± 3). The mean vessel volumes in pulmonary peels at 15-, 30-, and 45-mm depths were higher in pulmonary arterial hypertension (PAH) and PH secondary to lung disease compared with chronic thromboembolic PH (45-mm peel, mean difference: 6.4 mL [95% CI: 1, 11] [*P* < .001] vs 6.8 mL [95% CI: 1, 12] [*P* = .01]). Mean small vessel volumes at a diameter of less than 2 mm were lower in PAH and PH associated with left heart disease compared with controls (1.6-mm vessels, mean difference: –4.3 mL [95% CI: –8, –0.1] [*P* = .03] vs –6.8 mL [95% CI: –11, –2] [*P* < .001]). In patients with PH, the most significant positive correlation was noted with forced vital capacity percentage predicted (*r* = 0.30–0.40 [all *P* < .001] for TSPVVs and *r* = 0.21–0.25 [all *P* < .001] for PPVVs).

**Conclusion:**

The volume of pulmonary small vessels is reduced in pulmonary arterial hypertension and pulmonary hypertension (PH) associated with left heart disease, with similar volume of peel vessels compared with controls. For chronic thromboembolic PH, the volume of peel vessels is reduced. In PH, small pulmonary vessel volume is associated with pulmonary function tests.

Clinical trial registration no. NCT02565030

Published under a CC BY 4.0 license

*Online supplemental material is available for this article.*

## Graphical abstract

**Figure:**
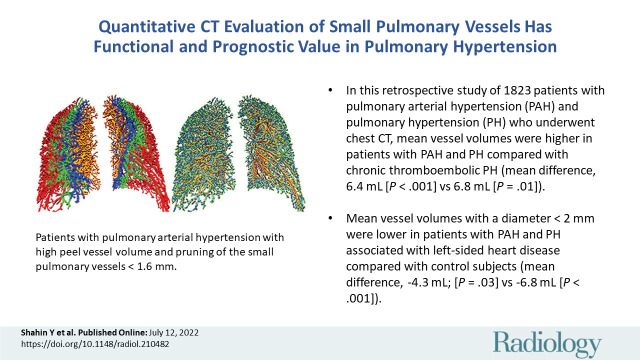

**Summary**

Quantitative CT assessment of small pulmonary vessels in pulmonary hypertension (PH) provides anatomic, physiologic, and prognostic insights, which might aid in the phenotyping of PH and in risk stratification.

**Key Results**

■ In this retrospective study of 1823 patients with pulmonary arterial hypertension (PAH) and pulmonary hypertension (PH) who underwent chest CT, the mean peel pulmonary vessel volumes were higher in patients with PAH and PH secondary to lung disease compared with chronic thromboembolic PH (mean difference, 6.4 mL [*P* < .001] vs 6.8 mL [*P* = .01]).
■ Mean small vessel volumes at a diameter of less than 2 mm were lower in patients with PAH and PH associated with left heart disease compared with controls (mean difference, –4.3 mL [*P* = .03] vs –6.8 mL [*P* < .001]).

## Introduction

Pulmonary hypertension (PH) is a condition of varied origin and defined by elevated mean pulmonary arterial pressure (mPAP) (≥20 mm Hg at rest) (1,2). PH has five major subtypes: group 1, pulmonary arterial hypertension (PAH); group 2, PH owing to left heart disease; group 3, PH associated with lung disease; group 4, chronic thromboembolic PH (CTEPH); and group 5, a miscellaneous group. PAH is characterized by remodeling of the small pulmonary arteries, with thickening of the intimal or medial layer of muscular vessel wall and proliferation of cells with smooth muscle expression causing distal remodeling (3,4). Evidence suggests that remodeling in many instances of PH is an outward process without luminal encroachment and might be due to failure of arterial relaxation or expansion (5–7).

The in vivo relationship between the morphologic change in small pulmonary vessels and PH is not well understood. Previous histologic studies showed that remodeling of small pulmonary vessels in PH is associated with decreased distensibility, leading to increased pulmonary vascular resistance and pulmonary arterial pressure. This led to studies on quantitative small pulmonary vessel assessment using multidetector CT mainly in patients with PH secondary to chronic obstructive pulmonary disease (COPD) (8–11) and in those with idiopathic pulmonary fibrosis (12). Automatic three-dimensional extraction of pulmonary vessels from multidetector CT has also been used to assess the severity of PH when pulmonary vessel volume could be measured in a lung peel (defined as the thickness of a lung section from the surface) or based on vessel diameter. However, quantitative multidetector CT assessment of small pulmonary vessels in different subtypes of PH compared with controls and how the pattern of pulmonary vascular involvement differs between PH subgroups has, to our knowledge, not been studied. We hypothesized that the morphologic change in small pulmonary vessels can be quantitatively evaluated with use of CT and that it adds prognostic value in PH. Therefore, the primary objective of this study was to quantitatively assess peel pulmonary vessel volumes (PPVVs) and small pulmonary vessel volumes (SPVVs) as estimated from CT pulmonary angiography (CTPA) scans in different subtypes of PH compared with controls. Secondary objectives were to study the relationship between PPVV, SPVV, pulmonary function, and right heart catheter metrics and to evaluate the prognostic value of PPVV and SPVV in PH.

## Materials and Methods

### Patients

**Figure 1:**
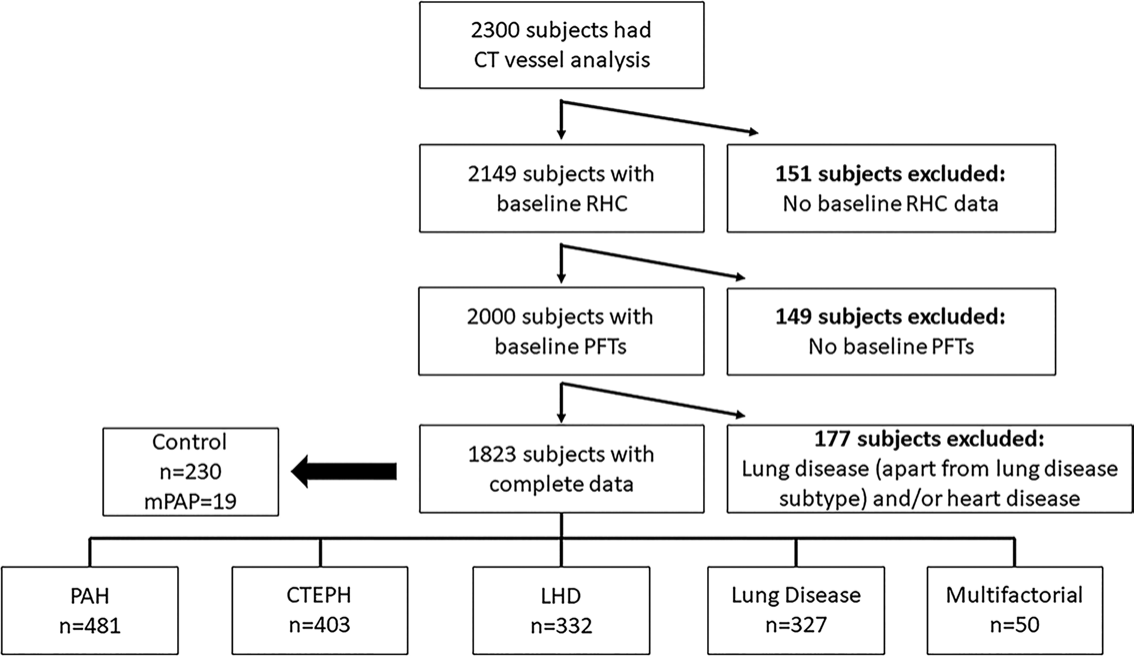
Study flowchart. Mean pulmonary arterial pressure (mPAP) is provided in millimeters of mercury. CTEPH = chronic thromboembolic pulmonary hypertension, LHD = left heart disease, PAH = pulmonary arterial hypertension, PFTs = pulmonary function tests, RHC = right heart catheter.

Consecutive patients diagnosed with PH who underwent right heart catheterization between January 2008 and February 2018 were prospectively recorded in Sheffield Pulmonary Vascular Unit databases as part of the ASPIRE (or Assessing the Spectrum of Pulmonary Hypertension Identified at a Referral Centre; *ClinicalTrials.gov* identifier NCT02565030) registry, as previously described (13,14). Patients’ demographic, imaging, and clinical metrics with follow-up data were prospectively collected using a census date of January 30, 2020. Right heart catheterization and pulmonary function metrics were included. Pulmonary function metrics were adjusted for age and sex and presented as percentage predicted. The study flowchart is presented in Figure 1.

The diagnosis of PH required patients to have right heart catheterization with an mPAP of 25 mm Hg or greater at rest (1). All patients were followed up until the date of death or census date. Patients referred for suspected PH and eventually found not to have PH after undergoing right heart catheterization, pulmonary function testing, and CTPA were considered the control group. Ethical approval for this study was granted by the North Sheffield Ethics Committee and Review Board (ref c06/Q2308/8). Informed consent was waived by the institutional review board because this was a secondary analysis of retrospective data.

### CTPA Image Acquisition and SPVV Analysis

CTPA examinations were performed on a 64-section multidetector CT scanner (LightSpeed, General Electric Medical Systems) in 1472 patients (acquisition parameters: 120 kV; 100 mA with autodose reduction; pitch, 1; rotation time, 0.5 second; field of view, 400 × 400 mm; and section thickness, 0.625 mm with a standard kernel filter) or a 320 detector-row CT system (Aquilion ONE/ViSION edition, Toshiba Medical Systems) in 351 patients (acquisition parameters: 120 kV; modulated mA with adaptive iterative dose reduction [15]; pitch-standard pitch factor, 0.813; helical pitch, 65; rotation time, 0.275 second; field of view, 500 mm; and section thickness, 0.5 mm with kernel filter code FC08). Contrast material was injected for 25 seconds via an antecubital vein with use of a weight-adapted injection protocol. Scanning was initiated 3 and 14 seconds after the attenuation in the region of interest placed in the pulmonary artery reached the threshold of 100 HU under a single breath hold.

J.T. is employed by VIDA Diagnostics and performed the software analysis of CT scans. Y.S. and A.J.S., who are not employed by industry, had control of the data and conducted the analysis. The authors did not receive industry funding.

**Figure 2:**
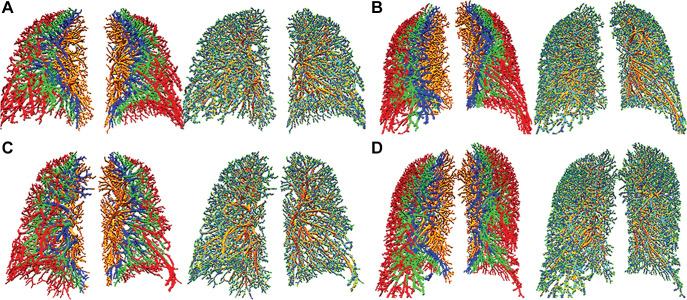
Vascular masks for peel pulmonary vessels (left pair of each image) show peel vessels at 15-mm (red), 30-mm (green), and 45-mm (dark blue) depths from pleural surface and small pulmonary vessels (right pair of each image) with a diameter of 0.4 mm (red), 0.8 mm (green), 1.2 mm (dark blue), 1.6 mm (yellow), and 2 mm (cyan) in **(A)** controls and **(B)** patients with pulmonary arterial hypertension with high peel vessel volume and pruning of the small pulmonary vessels less than 1.6 mm. **(C)** Chronic thromboembolic pulmonary hypertension vascular masks show attenuation of small pulmonary vessels and reduction of peel vessels. **(D)** Vascular masks for pulmonary hypertension secondary to left heart disease show pruning of the small pulmonary vessels and high peel vessel volume. The light brown color represents large proximal vessels.

**Figure 3:**
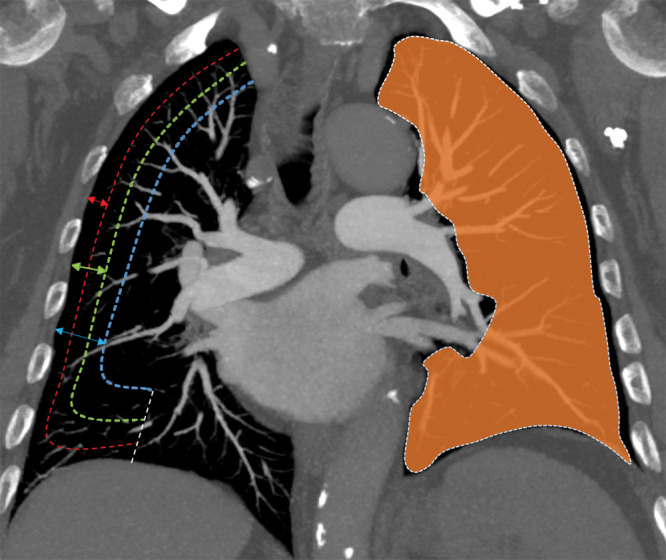
Schematic illustration on a coronal CT pulmonary angiography section shows the peel area concept at 15-mm (red), 30-mm (green), and 45-mm (blue) depths from pleural surface, as demonstrated on the right lung, and the area for measurement of small pulmonary vessels (orange), as demonstrated on the left lung.

PPVV and SPVV analysis was performed automatically, and vascular masks were checked by thoracic radiologists (Y.S. and A.J.S, with 7 and 20 years of experience, respectively) using Food and Drug Administration–approved lung quantitative imaging software (LungPrint, VIDA Diagnostics). This dedicated software was used to segment the lungs (16,17) and the pulmonary vessels automatically with visual confirmation by using a previously described approach (18–21) with high reproducibility, producing an intraclass correlation coefficient of 1 on replicate readings (19). Total SPVV (TSPVV) (in milliliters) of each segment was measured as the volume of detectable arteries and veins, including vessel walls and luminal blood. Total lung volume (in milliliters) was the combined volumes of left and right lungs. Total vessel volume (in milliliters) was the total vascular volume combined (arteries and veins). The vascular mask files were resampled to an isotropic voxel size of 0.2 mm^3^ to allow for a comparison between scans acquired at different resolutions. TSPVV metrics represented the volume taken up by small vessels (arteries and veins combined) and were corrected according to body surface area. TSPVV was calculated for vessels measuring 0.4, 0.8, 1.2, 1.6, and 2 mm in diameter (19). The PPVV (in milliliters) was the volume of blood vessels combined (arteries and veins). The suffix (ie, 15, 30, or 45) represents the thickness of the peel measured from the margin of the lungs at 15, 30, and 45 mm (Figs 2, 3). Vessel parameters were then adjusted according to body surface area.

### Right Heart Catheterization

Right heart catheterization was performed using a balloon-tipped 7.5-F thermodilution catheter (Becton Dickinson). Right heart catheterization was usually performed via the internal jugular vein with use of a Swan-Ganz catheter. Features of right heart catheterization required to define PH were mPAP of 25 mm Hg or greater at rest, with a pulmonary capillary wedge pressure of 15 mm Hg or less (1). Pulmonary vascular resistance was determined as follows: pulmonary vascular resistance = (mPAP – pulmonary capillary wedge pressure)/cardiac output. Cardiac output was measured with use of the thermodilution technique.

### Statistical Analysis

Normality of all variables was tested using the Kolmogorov-Smirnov test and histograms. Continuous variables were expressed as means ± SDs for parametric data and medians with IQRs for nonparametric data. Categorical data were presented as numbers of patients and percentages. Continuous parametric variables were compared using one-way analysis of variance with Bonferroni correction and Kruskal-Wallis and Mann-Whitney *U* tests for nonparametric variables. Categorical variables were compared using the Pearson χ^2^ test. Pearson correlation and linear regression were performed to determine the relationship between TSPVV, PPVV, and right heart catheterization and pulmonary function metrics, in which the dependent variable was log-transformed to overcome the effect of heteroscedasticity. Receiver operating characteristic analysis was used to determine prognostic volume thresholds for TSPVV in vessels with a diameter of 1.2 mm or less (TSPVV_≤1.2 mm_) and TSPVV in vessels with a diameter greater than 1.2 mm (TSPVV_>1.2 mm_). Survival was calculated from the date of the CT examination to date of death or census date with use of Kaplan-Meier plots and compared using the log-rank test. Univariable Cox proportional hazard regression analysis was used to assess the prognostic value of TSPVV_≤1.2 mm_ and TSPVV_>1.2 mm_ in addition to demographics, right heart catheterization, and pulmonary function metrics. Multivariable Cox proportional hazard regression analysis using the forward stepwise likelihood ratio method was performed for significant variables (*P* < .05) at univariable analysis and were previously reported as predictors of mortality in PH (22) (age, sex, mean right atrial pressure, pulmonary vascular resistance, venous oxygen saturation, transfer factor of the lung for carbon monoxide [TLco]) in addition to vessel analysis metrics.

All statistical tests were two sided, and *P* < .05 was considered indicative of statistically significant difference. SPSS for Windows, version 26 (IBM) was used for statistical analysis, and GraphPad Prism, version 8.3.0 (GraphPad Software) was used for presentation of data.

## Results

### Patients

Overall, 1823 patients (mean age, 69 years ± 13 [SD]; 1192 women [65%]) were analyzed. A total of 1593 patients with PH (mPAP, 43 mm Hg ± 13) were compared with 230 patient controls (mPAP, 19 mm Hg ± 3). Patients in the PH group had higher pulmonary artery systolic and diastolic pressures (all *P* < .001). They also had higher pulmonary capillary wedge pressure and pulmonary vascular resistance compared with controls (both *P* < .001). Arterial oxygen saturation and venous oxygen saturation were higher in the control group (both *P* < .001), as were all the pulmonary function metrics (all *P* < .001). Patients’ characteristics based on PH subtype compared with controls are summarized in Table 1. In brief, patients in the lung disease group were younger compared with PAH and left heart disease subtypes groups (*P* < .001). More women were in the PAH subtype group compared with CTEPH, lung disease, and multifactorial subtypes groups (*P* < .001). The control group had a higher number of patients with World Health Organization functional class I or II compared with all PH subtype groups (all *P* < .001). Patients in the CTEPH and left heart disease subtypes groups had the highest mPAP and pulmonary capillary wedge pressure mean values, respectively, compared with other subtypes groups (all *P* < .001).

**Table 1:**
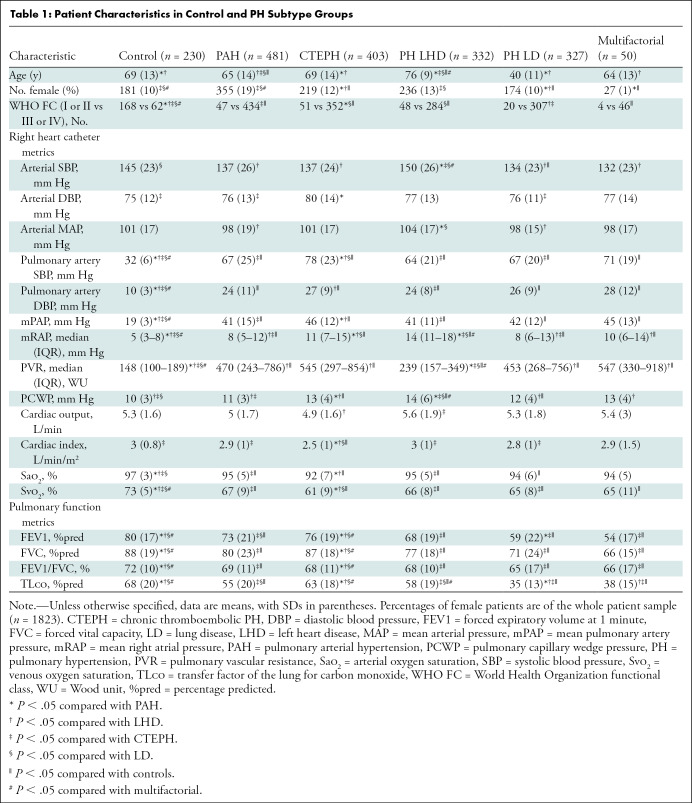
Patient Characteristics in Control and PH Subtype Groups

### Vessel Analysis

**Figure 4:**
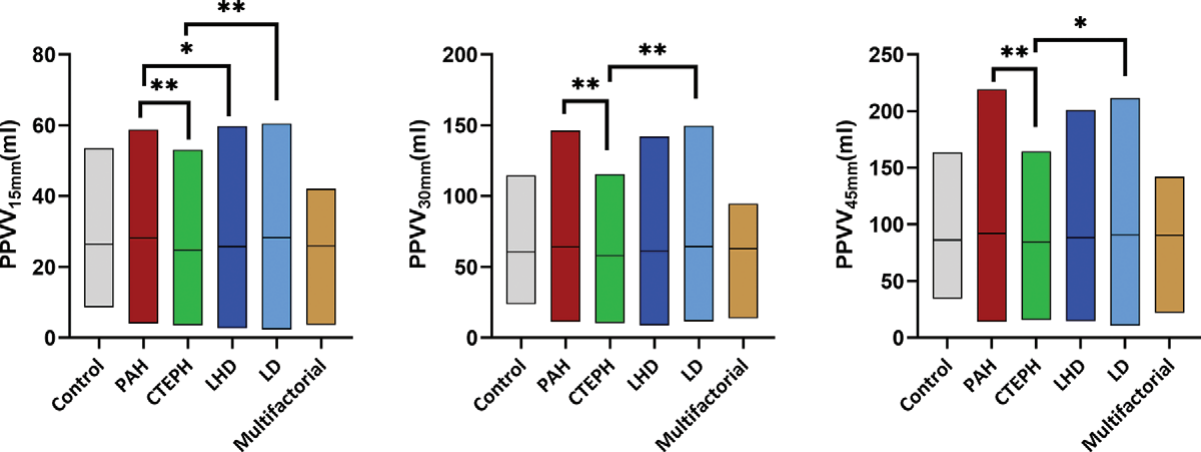
Floating bar charts show peel pulmonary vessel volumes in the control group and pulmonary hypertension subtypes. Bars represent peel vessel volumes, with the top and bottom edges showing minimum and maximum values. The horizontal line within each bar represents the mean. * = *P* < .05, ** = *P* < .001. CTEPH = chronic thromboembolic pulmonary hypertension, LD = lung disease, LHD = left heart disease, PAH = pulmonary arterial hypertension, PPVV_15 mm_ = peel pulmonary vessel volume within 15 mm from the lung surface, PPVV_30 mm_ = peel pulmonary vessel volume within 30 mm from the lung surface, PPVV_45 mm_ = peel pulmonary vessel volume within 45 mm from the lung surface.

***PPVV.—***Figure 4 shows vessel volumes within 15-, 30-, and 45-mm peel area in the control group and PH subtypes. We did not observe evidence of a difference in PPVVs at 15-, 30-, and 45-mm depths between the control group and all PH subtypes. Patients with PAH subtype had higher vessel volumes at 15-mm and 30-mm peels (mean difference, 3.4 mL [95% CI: 2, 5] and 6 mL [95% CI: 2, 10], respectively; both *P* < .001) and at 45 mm (mean difference, 6.4 mL [95% CI: 1, 11]; *P* < .001) compared with those with the CTEPH subtype. Vessel volumes were also higher at 15-mm and 30-mm peels (mean difference, 3.2 mL [95% CI: 1, 5] and 6 mL [95% CI: 2, 10], respectively; both *P* < .001) and at 45 mm (mean difference, 6.8 mL [95% CI: 1, 12]; *P* = .01) in the lung disease subtype compared with CTEPH.

***TSPVV.—***Figure E1 (online) shows mean volumes of small vessels of 0.4-, 1.2-, and 1.6-mm diameter in the control group and PH subtypes. Figure E2 (online) shows the mean volumes of small vessels of 0.8- and 2-mm diameter. The control group had a higher volume of small vessels of less than 2 mm in diameter compared with the PAH subtype (mean difference, 0.4 mL [95% CI: 0.1, 0.7] [*P* < .001] for 0.4-mm diameter vessels vs 1.4 mL [95% CI: 0.2, 2.7] for 0.8-mm diameter vessels; mean difference: 3 mL [95% CI: 0.3, 6] for 1.2-mm diameter vessels and 4 mL [95% CI: 0.1, 8] for 1.6-mm vessels; all *P* = .03). The control group had a higher volume of small vessels of less than 2 mm in diameter (mean difference, 0.5 mL [95% CI: 0.2, 0.8] for 0.4-mm vessels; mean difference: 2 mL [95% CI: 1, 4] for 0.8-mm vessels; mean difference: 5 mL [95% CI: 2, 8] for 1.2-mm vessels; and mean difference: 6.8 mL [95% CI: 2, 11] for 1.6-mm vessels; all *P* < .001) and 2-mm diameter (mean difference, 7.6 mL [95% CI: 1.6, 13.6]; *P* = .003) compared with left heart disease subtype. All volumes of small vessels were higher in CTEPH compared with left heart disease (mean difference, 0.3 mL [95% CI: 0.2, 0.5] [*P* = .03] for 0.4-mm vessels vs 2 mL [95% CI: 0.6, 3] for 0.8-mm vessels; mean difference: 4 mL [95% CI: 2, 7] for 1.2-mm vessels; and mean difference: 6.5 mL [95% CI: 2.7, 10] for 1.6-mm vessels [all *P* < .001] and mean difference: 7 mL [95% CI: 2, 12] [*P* < .001] for 2-mm diameter vessels).

### Correlation with Pulmonary Function and Right Heart Catheter Metrics

Figure E3 (online) shows correlations of PPVV with forced vital capacity percentage predicted (FVC%pred). Figures E4 and E5 (online) illustrate correlations of TSPVV and total vessel volume in patients with PH with FVC%pred, and Figure E6 (online) shows correlations of TSPVV and total vessel volume with forced expiratory volume at 1 minute percentage predicted (FEV1%pred). Modest positive correlations were noted between all volumes of peel vessels and FVC%pred (*r* = 0.21–0.25 [all *P* < .001]) and mild positive correlations with FEV1%pred. No significant correlation was noted with TLcopercentage predicted (TLco%pred). Moderate to strong positive correlations were noted between all volumes of small vessels and FVC%pred (*r* = 0.30–0.40 [*P* < .001]) (Figs E4, E5 [online]) and mild positive correlations with FEV1%pred (Fig E6 [online]). There was modest positive correlation with TLco%pred (*r* = 0.15–0.22; all *P* < .001). Correlations between TSPVV, PPVV, total vessel volume in all PH subtypes, and pulmonary function metrics are summarized in Table E1 (online).

Vessel volumes at 30-mm and 45-mm peels showed mild positive correlation with cardiac output and cardiac index (*r* = 0.09–0.13 for 30-mm peel and *P* = .006 for 45-mm peel [*P* < .001]). All volumes of small vessels (0.4- to 2-mm diameter) showed a mild negative correlation with pulmonary capillary wedge pressure and mean right atrial pressure (*r* = 0.10–0.19, *P* < .001 for 0.4-, 0.8-, 1.2-, and 1.6-mm diameter vessels and *P* = .004 for 2-mm vessels). There was no significant correlation observed between vessel volumes and mPAP.

### Prognostic Value of Vessel Analysis

**Figure 5:**
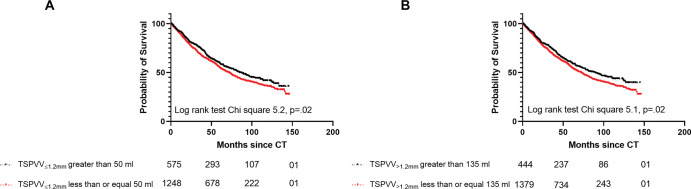
Kaplan-Meier survival analysis from date of CT examination shows the outcome of **(A)** patients with pulmonary hypertension based on total small pulmonary vessel volume at a diameter of 1.2 mm or less (TSPVV_≤1.2 mm_) of greater than 50 mL and TSPVV_≤1.2 mm_ of 50 mL or less and **(B)** patients with total small pulmonary vessel volume at a diameter greater than 1.2 mm (TSPVV_>1.2 mm_) of greater than 135 mL and TSPVV_>1.2 mm_ of 135 mL or less. Numbers at risk for each group are presented below the plot.

Prognostic volume thresholds of 50 and 135 mL were identified using receiver operating characteristic analysis for TSPVV_≤1.2 mm_ and TSPVV_>1.2 mm_, respectively. As shown in Figure 5, median survival for TSPVV_≤1.2 mm_ greater than 50 mL was longer than that for TSPVV_≤1.2 mm_ of 50 mL or less (85 vs 71 months [95% CI: 73, 97]; *P* = .02). Median survival for TSPVV_>1.2 mm_ greater than 135 mL was longer than that of TSPVV_>1.2 mm_ of 135 mL or less (88 vs 72 months [95% CI: 73, 98]; *P* = .02). Results of the univariable and multivariable Cox regression hazard analyses for the PH group are summarized in Table 2. TSPVV_≤1.2 mm_ greater than 50 mL (hazard ratio = 0.85; *P* = .02) and TSPVV_>1.2 mm_ greater than 135 mL (hazard ratio = 0.84; *P* = .02) were significant predictors of mortality at univariable analysis. TSPVV_≤1.2 mm_ greater than 50 mL (hazard ratio = 0.79; *P* = .04) was an independent predictor of mortality at multivariable analysis in addition to age, male sex, mean right atrial pressure, pulmonary vascular resistance, and venous oxygen saturation.

**Table 2:**
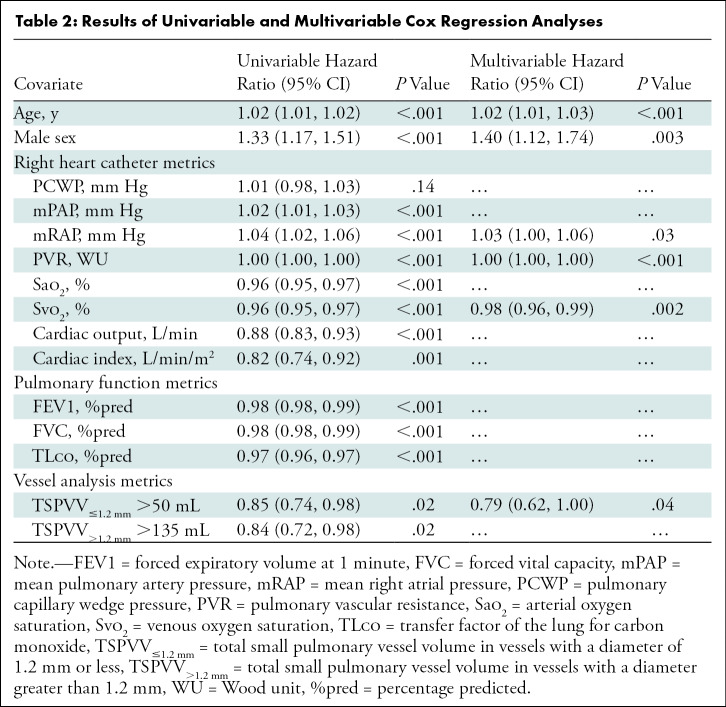
Results of Univariable and Multivariable Cox Regression Analyses

### Subgroup Analysis

***Incident patients.—***To assess vessel volume differences in patients who were treatment-naive (without any treatment effect), a subgroup analysis was performed for incident cases (patients who underwent CTPA before the diagnostic visit). A total of 1209 patients (age 69 years ± 13; 798 women [66%]) were analyzed. A total of 1036 patients (subgroup: PAH, 283 [27%]; CTEPH, 259 [25%]; left heart disease, 224 [22%]; lung disease, 231 [22%]; multifactorial, 39 [4%]) with PH (mPAP, 43 mm Hg ± 12) were compared with 173 controls (mPAP, 19 mm Hg ± 3).

There was no evidence of a difference in the PPVV between the control group and all PH subtypes. The control group had a higher SPVV (0.4- to 2-mm diameter) compared with left heart disease subtype (*P* < .001 for 0.4-, 0.8-, 1.2-, and 1.6-mm diameter vessels and *P* = .004 for 2-mm vessels). Small vessels (0.8–2-mm diameter) were higher in CTEPH compared with left heart disease (all *P* < .001).

***Chronic thromboembolic disease distribution.—***A subgroup analysis was conducted to assess the impact of chronic thromboembolic disease distribution on vessel volumes, which was available for 300 patients (proximal disease, *n* = 100; distal disease, *n* = 118; and mixed disease, *n* = 82) in the CTEPH group. There was no evidence of a difference in PPVV (at 15-, 30-, and 45-mm depths) and SPVV (0.4- to 2-mm diameter) in patients with proximal compared with distal or mixed disease distribution (*P* > .05).

## Discussion

**Figure 6:**
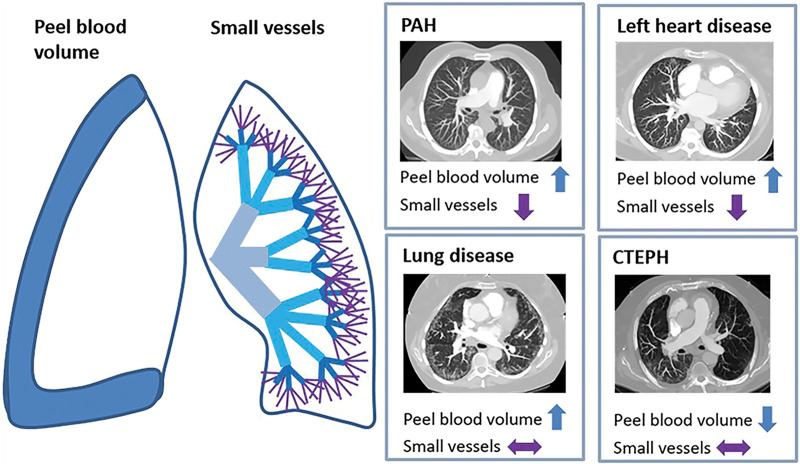
Schematic illustration of the changes in the pulmonary peel and small vessels seen at quantitative CT for the various causes of elevated pulmonary arterial pressure. The dark-blue area drawn on the right lung represents the peel vessel volume, and the purple lines drawn on the left lung represent an example of the small vessels. Changes in peel volume in the left heart disease and pulmonary arterial hypertension (PAH) groups are compared with the chronic thromboembolic pulmonary hypertension (CTEPH) group. All other changes are compared with the control group.

In this secondary chest quantitative CT analysis of 1593 patients with various causes of elevated pulmonary artery pressure (pulmonary arterial hypertension [PAH], chronic thromboembolic pulmonary hypertension [CTEPH], pulmonary hypertension [PH] due to left heart disease, PH due to lung disease, or multifactorial causes) and 230 controls, we found that the mean vessel volumes in pulmonary peels at 15-, 30-, and 45-mm depths were higher in patients with PAH and PH secondary to lung disease compared with CTEPH. Mean small vessel volumes at a diameter of less than 2 mm were lower in PAH and PH associated with left heart disease compared with controls. In patients with PH, the most significant positive correlation was noted between all total small pulmonary vessel volumes (TSPVVs) and forced vital capacity percentage predicted, followed by forced expiratory volume at 1 minute percentage predicted and transfer factor of the lung for carbon monoxide percentage predicted. In addition, TSPVV was lower in patients with PAH compared with controls and patients with CTEPH. It was also higher in patients with no PH and in CTEPH compared with PH secondary to left heart disease. The study also highlights that higher TSPVV and peel pulmonary vessel volume measured at CT pulmonary angiography are associated with better pulmonary function in patients with PH; however, there was no association with pulmonary arterial pressure. A summary of changes in volume of peel and small pulmonary vessels is illustrated in Figure 6.

PAH is associated with vascular remodeling, which involves medial hypertrophy of the muscular and elastic arteries and proliferation of cells of smooth muscle expression. Dilatation and intimal atheromas of the elastic pulmonary arteries and veno-occlusive lesions without an associated arteriopathy have also been found at histologic examination (23–25). In this study, PPVV was higher in patients with PAH compared with those with CTEPH. This may be explained by dilatation of elastic pulmonary vessels in the peel of the lung due to vascular remodeling and luminal narrowing of downstream small pulmonary arteries. TSPVV was lower compared with controls, suggesting coexistent pruning of small blood vessels at thresholds of less than 1.6 mm in diameter. In contrast, our study showed that in CTEPH, vascular changes from clot burden, which include arterial narrowing or occlusion and webs, result in an overall lower blood volume in the peel of the lung but show higher blood volume in small pulmonary arteries (26,27). We postulate this is because of a greater proportion of smaller pulmonary vessels due to proximal obstructions, leading to downstream attenuation of vessels within the related segments of the pulmonary tree.

TSPVV in patients with PH secondary to left heart disease was lower than in patients CTEPH and the control group. This may indicate pruning of small pulmonary arteries akin to PAH; remodeling of small pulmonary arteries and veins is evident in PH associated with left heart disease (28). In patients with PH secondary to lung disease, our results showed higher PPVV compared with those with CTEPH, consistent with CTEPH having loss of peripheral blood volume. In contrast, TSPVV did not show significant difference compared with other groups. Previous studies demonstrated significant alterations at the microvascular level in patients with COPD (8–11,29–33). Further studies comparing COPD and interstitial lung disease subtypes are required to further characterize vessel changes in patients with PH associated with lung disease.

Hueper et al (31) showed a 38% reduction in pulmonary microvascular volume in patients with mild COPD and 53% in those with severe COPD compared with patients without COPD, suggestive of marked microvascular damage. However, the heterogeneity of the lung disease group in our study, including patients with COPD in addition to lung fibrosis, might have been a confounding factor when evaluating TSPVV. Additionally, the severity of lung disease in our sample varied between patients. Microvascular damage is best seen in patients with severe COPD (31).

Significant positive correlations were noted between TSPVV and pulmonary function metrics. The strongest correlations were observed between PPVV and TSPVV and forced vital capacity, which was most apparent in the PAH and lung disease subtypes. Additionally, there were mild to moderate correlations with forced expiratory volume at 1 minute and TLco. Our study showed that higher vessel volume is associated with better pulmonary function. There was no evidence of a correlation between SPVVs and mPAP in our study, which is in agreement with other studies (29,34). However, Matsuoka et al (9) found significant correlations between mPAP and pulmonary vessels in a cross-sectional area of less than 5 mm^2^ in a small cohort of patients with severe emphysema. Their study was limited by the small number of patients and the assessment of pulmonary vessels with use of the cross-sectional area technique, which can be influenced by automatic exposure control from the scanner and by patient position.

Previous studies showed the prognostic value of quantitated vessel volumes in patients with lung fibrosis (12,35). In PH, several studies reported prognostic value of pulmonary artery diameter but not SPVV (36–38). Our study is the first, to our knowledge, to demonstrate the prognostic value of SPVVs in PH with higher SPVV associated with decreased mortality. In particular, TSPVV_≤1.2 mm_ greater than a threshold of 50 mL is independently associated with decreased mortality. An increase of TSPVV_≤1.2 mm_ by 1 mL above the 50 mL threshold was associated with a decrease in mortality by a factor of 0.79.

Our study has limitations. This is a secondary analysis of prospectively collected data, which makes it difficult to draw definitive conclusions beyond the variability in SPVV between PH subtypes and the association of SPVV with pulmonary function metrics. Additionally, the quantitative CT analysis included both arteries and veins. This is a recognized limitation of this technology. Some early studies have shown success, and further research in different disease groups is required to validate these promising approaches (39–41). The sample comprised patients who were scanned with two different scanners with different section thicknesses (0.625 mm and 0.5 mm); however, no significant difference in vessel volumes was detected at analysis. The control group consisted of patients with suspected PH with comorbidities and elevated mPAP compared with individuals without PH. However, our results showed differences between the groups in a real-world setting. The depth of inspiration on these quantitative CT examinations may impact these measures of SPVV, and the repeatability and precision of these metrics has not been determined.

In conclusion, quantitative CT assessment of small pulmonary vessel volume (SPVV) provides anatomic and physiologic insights that may aid image-based phenotyping and risk stratification in pulmonary hypertension (PH). Future studies should focus on evaluating treatment effects on SPVV and on stratifying patients for treatment based on SPVV to improve outcomes in PH.

## References

[1] Galiè N , Humbert M , Vachiery JL , et al. 2015 ESC/ERS Guidelines for the diagnosis and treatment of pulmonary hypertension: the Joint Task Force for the Diagnosis and Treatment of Pulmonary Hypertension of the European Society of Cardiology (ESC) and the European Respiratory Society (ERS): Endorsed by: Association for European Paediatric and Congenital Cardiology (AEPC), International Society for Heart and Lung Transplantation (ISHLT). Eur Respir J 2015;46(4):903–975. [Published correction appears in Eur Respir J 2015;46(6):1855-1856.] 26318161 10.1183/13993003.01032-2015

[2] Simonneau G , Montani D , Celermajer DS , et al. Haemodynamic definitions and updated clinical classification of pulmonary hypertension. Eur Respir J 2019;53(1):1801913. 30545968 10.1183/13993003.01913-2018 PMC6351336

[3] Voelkel NF , Tuder RM . Hypoxia-induced pulmonary vascular remodeling: a model for what human disease? J Clin Invest 2000;106(6):733–738. 10995781 10.1172/JCI11144 PMC381402

[4] Stenmark KR , Meyrick B , Galie N , Mooi WJ , McMurtry IF . Animal models of pulmonary arterial hypertension: the hope for etiological discovery and pharmacological cure. Am J Physiol Lung Cell Mol Physiol 2009;297(6):L1013–L1032. 19748998 10.1152/ajplung.00217.2009

[5] Hyvelin JM , Howell K , Nichol A , Costello CM , Preston RJ , McLoughlin P . Inhibition of Rho-kinase attenuates hypoxia-induced angiogenesis in the pulmonary circulation. Circ Res 2005;97(2):185–191. 15961717 10.1161/01.RES.0000174287.17953.83

[6] Stenmark KR , McMurtry IF . Vascular remodeling versus vasoconstriction in chronic hypoxic pulmonary hypertension: a time for reappraisal? Circ Res 2005;97(2):95–98. 16037575 10.1161/01.RES.00000175934.68087.29

[7] McMurtry IF , Abe K , Ota H , Fagan KA , Oka M . Rho kinase-mediated vasoconstriction in pulmonary hypertension. Adv Exp Med Biol 2010;661:299–308. 20204738 10.1007/978-1-60761-500-2_19

[8] Muñoz-Esquerre M , López-Sánchez M , Escobar I , et al. Systemic and pulmonary vascular remodelling in chronic obstructive pulmonary disease. PLoS One 2016;11(4):e0152987. 27046203 10.1371/journal.pone.0152987 PMC4821623

[9] Matsuoka S , Washko GR , Yamashiro T , et al. Pulmonary hypertension and computed tomography measurement of small pulmonary vessels in severe emphysema. Am J Respir Crit Care Med 2010;181(3):218–225. 19875683 10.1164/rccm.200908-1189OC PMC2817812

[10] Yoshimura K , Suzuki Y , Uto T , Sato J , Imokawa S , Suda T . Morphological changes in small pulmonary vessels are associated with severe acute exacerbation in chronic obstructive pulmonary disease. Int J Chron Obstruct Pulmon Dis 2016;11:1435–1445. 27418816 10.2147/COPD.S107424 PMC4934566

[11] Coste F , Dournes G , Dromer C , et al. CT evaluation of small pulmonary vessels area in patients with COPD with severe pulmonary hypertension. Thorax 2016;71(9):830–837. 27084957 10.1136/thoraxjnl-2015-207696

[12] Jacob J , Bartholmai BJ , Rajagopalan S , et al. Mortality prediction in idiopathic pulmonary fibrosis: evaluation of computer-based CT analysis with conventional severity measures. Eur Respir J 2017;49(1):1601011. 27811068 10.1183/13993003.01011-2016

[13] Hurdman J , Condliffe R , Elliot CA , et al. ASPIRE registry: assessing the Spectrum of Pulmonary hypertension Identified at a REferral centre. Eur Respir J 2012;39(4):945–955. 21885399 10.1183/09031936.00078411

[14] Quadery SR , Swift AJ , Billings CG , et al. The impact of patient choice on survival in chronic thromboembolic pulmonary hypertension. Eur Respir J 2018;52(3):1800589. 30002102 10.1183/13993003.00589-2018 PMC6340636

[15] Kiely DG , Levin D , Hassoun P , et al. EXPRESS: Statement on imaging and pulmonary hypertension from the Pulmonary Vascular Research Institute (PVRI). Pulm Circ 2019;9(3):1–32. 10.1177/2045894019841990 PMC6732869 30880632

[16] Schuhmann M , Raffy P , Yin Y , et al. Computed tomography predictors of response to endobronchial valve lung reduction treatment. Comparison with Chartis. Am J Respir Crit Care Med 2015;191(7):767–774. 25635349 10.1164/rccm.201407-1205OC

[17] Valipour A , Shah PL , Gesierich W , et al. Patterns of emphysema heterogeneity. Respiration 2015;90(5):402–411. 26430783 10.1159/000439544 PMC4756593

[18] Iyer KS , Newell JD Jr , Jin D , et al. Quantitative dual-energy computed tomography supports a vascular etiology of smoking-induced inflammatory lung disease. Am J Respir Crit Care Med 2016;193(6):652–661. 26569033 10.1164/rccm.201506-1196OC PMC4824939

[19] Aaron CP , Hoffman EA , Lima JAC , et al. Pulmonary vascular volume, impaired left ventricular filling and dyspnea: the MESA lung study. PLoS One 2017;12(4):e0176180. 28426728 10.1371/journal.pone.0176180 PMC5398710

[20] Shikata H , McLennan G , Hoffman EA , Sonka M . Segmentation of pulmonary vascular trees from thoracic 3D CT images. Int J Biomed Imaging 2009;2009:636240. 20052391 10.1155/2009/636240 PMC2801012

[21] Aaron CP , Hoffman EA , Kawut SM , et al. Ambient air pollution and pulmonary vascular volume on computed tomography: the MESA Air Pollution and Lung cohort studies. Eur Respir J 2019;53(6):1802116. 31167881 10.1183/13993003.02116-2018 PMC6910868

[22] Benza RL , Miller DP , Gomberg-Maitland M , et al. Predicting survival in pulmonary arterial hypertension: insights from the Registry to Evaluate Early and Long-Term Pulmonary Arterial Hypertension Disease Management (REVEAL). Circulation 2010;122(2):164–172. 20585012 10.1161/CIRCULATIONAHA.109.898122

[23] Pietra GG , Capron F , Stewart S , et al. Pathologic assessment of vasculopathies in pulmonary hypertension. J Am Coll Cardiol 2004;43(12 Suppl S):25S–32S. 15194175 10.1016/j.jacc.2004.02.033

[24] Shimoda LA , Laurie SS . Vascular remodeling in pulmonary hypertension. J Mol Med (Berl) 2013;91(3):297–309. 23334338 10.1007/s00109-013-0998-0 PMC3584237

[25] Miura A , Nakamura K , Kusano KF , et al. Three-dimensional structure of pulmonary capillary vessels in patients with pulmonary hypertension. Circulation 2010;121(19):2151–2153. 20479166 10.1161/CIR.0b013e3181e037c1

[26] Hoeper MM , Mayer E , Simonneau G , Rubin LJ . Chronic thromboembolic pulmonary hypertension. Circulation 2006;113(16):2011–2020. 16636189 10.1161/CIRCULATIONAHA.105.602565

[27] Rahaghi FN , Ross JC , Agarwal M , et al. Pulmonary vascular morphology as an imaging biomarker in chronic thromboembolic pulmonary hypertension. Pulm Circ 2016;6(1):70–81. 27162616 10.1086/685081 PMC4860553

[28] Fayyaz AU , Edwards WD , Maleszewski JJ , et al. Global pulmonary vascular remodeling in pulmonary hypertension associated with heart failure and preserved or reduced ejection fraction. Circulation 2018;137(17):1796–1810. 29246894 10.1161/CIRCULATIONAHA.117.031608 PMC5915920

[29] Wright JL , Petty T , Thurlbeck WM . Analysis of the structure of the muscular pulmonary arteries in patients with pulmonary hypertension and COPD: National Institutes of Health nocturnal oxygen therapy trial. Lung 1992;170(2):109–124. 1501507 10.1007/BF00175982

[30] Hopkins N , McLoughlin P . The structural basis of pulmonary hypertension in chronic lung disease: remodelling, rarefaction or angiogenesis? J Anat 2002;201(4):335–348. 12430958 10.1046/j.1469-7580.2002.00096.x PMC1570922

[31] Hueper K , Vogel-Claussen J , Parikh MA , et al. Pulmonary microvascular blood flow in mild chronic obstructive pulmonary disease and emphysema. the MESA COPD study. Am J Respir Crit Care Med 2015;192(5):570–580. 26067761 10.1164/rccm.201411-2120OC PMC4595687

[32] Hale KA , Niewoehner DE , Cosio MG . Morphologic changes in the muscular pulmonary arteries: relationship to cigarette smoking, airway disease, and emphysema. Am Rev Respir Dis 1980;122(2):273–278. 7416604 10.1164/arrd.1980.122.2.273

[33] Wright JL , Lawson L , Paré PD , et al. The structure and function of the pulmonary vasculature in mild chronic obstructive pulmonary disease. The effect of oxygen and exercise. Am Rev Respir Dis 1983;128(4):702–707. 6625346 10.1164/arrd.1983.128.4.702

[34] Yamato H , Sun JP , Churg A , Wright JL . Guinea pig pulmonary hypertension caused by cigarette smoke cannot be explained by capillary bed destruction. J Appl Physiol (1985) 1997;82(5):1644–1653. 9134915 10.1152/jappl.1997.82.5.1644

[35] Jacob J , Bartholmai BJ , Rajagopalan S , et al. Predicting outcomes in idiopathic pulmonary fibrosis using automated computed tomographic analysis. Am J Respir Crit Care Med 2018;198(6):767–776. 29684284 10.1164/rccm.201711-2174OC PMC6222463

[36] Tonelli AR , Johnson S , Alkukhun L , Yadav R , Dweik RA . Changes in main pulmonary artery diameter during follow-up have prognostic implications in pulmonary arterial hypertension. Respirology 2017;22(8):1649–1655. 28514116 10.1111/resp.13073 PMC5884162

[37] Żyłkowska J , Kurzyna M , Florczyk M , et al. Pulmonary artery dilatation correlates with the risk of unexpected death in chronic arterial or thromboembolic pulmonary hypertension. Chest 2012;142(6):1406–1416. 22797193 10.1378/chest.11-2794

[38] Colin GC , Gerber BL , de Meester de Ravenstein C , et al. Pulmonary hypertension due to left heart disease: diagnostic and prognostic value of CT in chronic systolic heart failure. Eur Radiol 2018;28(11):4643–4653. 29761362 10.1007/s00330-018-5455-6

[39] Charbonnier JP , Brink M , Ciompi F , Scholten ET , Schaefer-Prokop CM , van Rikxoort EM . Automatic pulmonary artery-vein separation and classification in computed tomography using tree partitioning and peripheral vessel matching. IEEE Trans Med Imaging 2016;35(3):882–892. 26584489 10.1109/TMI.2015.2500279

[40] Nardelli P , Jimenez-Carretero D , Bermejo-Pelaez D , et al. Pulmonary artery-vein classification in CT images using deep learning. IEEE Trans Med Imaging 2018;37(11):2428–2440. 29993996 10.1109/TMI.2018.2833385 PMC6214740

[41] Pienn M , Burgard C , Payer C , et al. healthy lung vessel morphology derived from thoracic computed tomography. Front Physiol 2018;9:346. 29755360 10.3389/fphys.2018.00346 PMC5932382

